# Effects of Antidepressant Treatment on Neurotrophic Factors (BDNF and IGF-1) in Patients with Major Depressive Disorder (MDD)

**DOI:** 10.3390/jcm10153377

**Published:** 2021-07-30

**Authors:** Anna Mosiołek, Jadwiga Mosiołek, Sławomir Jakima, Aleksandra Pięta, Agata Szulc

**Affiliations:** 1 Department of Psychiatry, Faculty of Health Sciences, Medical University of Warsaw, Żwirki i Wigury 61 Street, 02-091 Warszawa, Poland; aleksandrapieta123@gmail.com (A.P.); agataszulc@poczta.onet.pl (A.S.); 2 Mazovia Specialist Health Center in Pruszków, Partyzantów 2/4 Street, 05-802 Pruszków, Poland; s.jakima@wp.pl; 3 Faculty of Medicine, Wroclaw Medical University, Wybrzeże Ludwika Pasteura 1 Street, 50-367 Wroclaw, Poland; mosiolek.jadwiga@gmail.com

**Keywords:** BDNF, IGF-1, antidepressants, neurotrophic factors

## Abstract

Major depressive disorder (MDD) remains the subject of ongoing research as a multifactorial disease and a serious public health problem. There is a growing body of literature focusing on the role of neurotrophic factors in pathophysiology of MDD. A neurotrophic hypothesis of depression proposes that abnormalities of neurotrophins serum levels lead to neuronal atrophy and decreased neurogenesis, resulting in mood disorders. Consequently, in accordance with recent findings, antidepressant treatment modifies the serum levels of neurotrophins and thus leads to a clinical improvement of MDD. The purpose of this review is to summarize the available data on the effects of various antidepressants on serum levels of neurotrophins such as brain-derived neurotrophic factor (BDNF) and insulin-like growth factor (IGF-1). In addition, the authors discuss their role as prognostic factors for treatment response in MDD. A literature search was performed using the PubMed database. Following the inclusion and exclusion criteria, nine original articles and three meta-analyses were selected. The vast majority of studies have confirmed the effect of antidepressants on BDNF levels. Research on IGF-1 is limited and insufficient to describe the correlation between different antidepressant drugs and factor serum levels; however, four studies indicated a decrease in IGF-1 after treatment. Preliminary data suggest BDNF as a promising predictor of treatment response in MDD patients. The role of IGF-1 needs further investigation.

## 1. Introduction

Depression is the most commonly diagnosed mental disorder [1]. Current estimates state that around 1 in 10 individuals suffer from depression at least once in a lifetime, which would require medical treatment [2]. Depression has a multifactorial etiology, and individual hypotheses related to its formation complement each other. In major depression we are dealing with symptoms of immune activation, oxidative stress, changes in immune reaction and activation of the inflammatory response [3,4,5,6,7,8,9,10,11,12,13,14,15]. The updated molecular hypothesis of depression postulates that the response to antidepressants is associated with intracellular mechanisms that influence neurotrophic factors necessary for the survival and function of specific neurons [16,17]. Brain-derived neurothropic factor (BDNF) is a growth factor synthesized in the cell bodies of neurons and in glia. It affects neuronal maturation, formation of synapses and synaptic plasticity. BDNF is also associated with the development of psychiatric disorders. According to the neurotrophic hypothesis of depression, the deficiency of BDNF and other growth factors may contribute to the atrophy of certain limbic structures, including the hippocampus and prefrontal cortex, observed in patients with depression, and antidepressant drugs act by increasing the levels of BDNF [18,19,20,21,22,23]. Neurotrophins, such as brain-derived neurotrophic factor (BDNF), are the main regulators of neuronal survival, growth and differentiation during the development. Signaling via BDNF and its receptor, tropomycin receptor kinase B (TrkB), plays a key role in the pathophysiology of depression and in the therapeutic mechanisms of antidepressants [24]. The mechanisms underlying the effectiveness of antidepressants may include an increase in BDNF, which is responsible for neuroplasticity in the nervous system [25,26]. Interestingly, antidepressant treatment has been found to increase BDNF levels in the hippocampus, which further justifies the importance of this neurotrophin in MDD [27]. Postmortem examination conducted on brain of suicide victims showed significantly lower levels of BDNF than those of control group [28]. Insulin-like growth factor (IGF-1) is a protein primarily produced by the liver and its secretion is stimulated by a growth hormone (GH). IGF-1 plays an important role in glucose metabolism, fat and glycogen synthesis, tissue and cell development, as well as immune processes. Moreover, IGF-1 has a wide range of functions in the central nervous system. It has been proven to act on the function of synapses, the metabolism of brain cells and to have neuroprotective properties [29,30]. It has potent neurotrophic and neurogenic effects. The activity of IGF-1 being modulated by the immune system is a very common hypothesis in the pathogenesis of depression [29,31]. Studies show that a decrease in the brain IGF-1 expression disrupts neuroplasticity mechanisms and promotes cerebral inflammatory pathways, leading to morphological deterioration of brain areas responsible for emotional and cognitive processing [32]. These relationships are being investigated in further studies, which suggest that abnormal IGF-1 activity may be associated with the development of mood disorders [31]. One interesting aspect is the synergic beneficial effect of the neurotrophins on neuronal survival and neuroplasticity. Recent evidence suggests that IGF-1 induces a significant increase in BDNF receptors (Trk-B) and enhances BDNF activity measured by ERK1/2 phosphorylation [33].

There is evidence of a modulating effect of antidepressants on depression via the neurotrophins in preclinical and clinical models of depression; however, the results are conflicting; therefore, the therapeutic role of antidepressants cannot be clearly stated [21,22,34,35,36,37,38,39,40,41,42,43,44,45,46]. The pharmacotherapy of depression most frequently used in clinical practice is based on the use of drugs that increase the level of monoamines. However, in about 30% of patients, such treatment is insufficiently effective or ineffective. For this reason, it is worth looking at how individual drugs affect the neurochemical changes observed in depression, which could lead to the development of more targeted treatment in the future. Assessment of the cure with the help of a biomarker could reduce ambiguity and give therapy a definite direction and lead to personalized treatment [38,47].

There are several major classes of antidepressants, such as tricyclic antidepressants (TCA), monoamine oxidase inhibitors (MAO), selective serotonin reuptake inhibitors (SSRI), selective serotonin and norepinephrine reuptake inhibitors (SNRI) and atypical antidepressants. SSRIs work by blocking the serotonin transporter, which inhibits 5-HT reuptake in the synaptic cleft [48]. Serotonin norepinephrine reuptake inhibitors (SNRIs) are a group of antidepressants that inhibit the reabsorption of two neurotransmitters, serotonin and noradrenaline. As a result, their concentration increases in the synaptic cleft between neurons, which results in an increase in neurotransmission from one nerve cell to another [49]. The aim of the study was to assess how antidepressants affect the level of neurotrophic markers and whether they could be a predictor of response to antidepressants [49,50].

## 2. Materials and Methods

### Search Strategy

Electronic databases (PubMed, Web of Science, Google Scholar) were systematically searched. All previous human studies through May 2021 (published original clinical studies, meta-analyses) comparing pre- and post-antidepressant treatment with peripheral BDNF and IGF levels were considered. The search terms were settled as follows: “neurotrophic factors”, “brain-derived neurotrophic factor” OR “BDNF”, “IGF-1”, “antidepressant” AND “major depression” OR “major depressive disorder” OR “MDD” OR “depressive episode” OR “depression”.

### Study Selection Strategy

**Figure 1:**
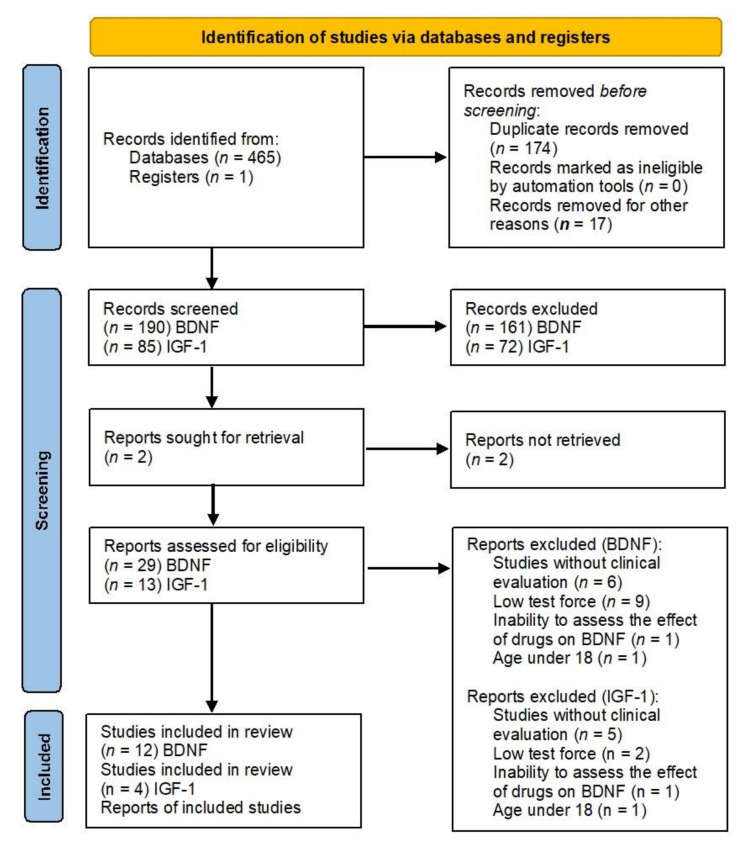
PRISMA flow diagram for our systematic review.

Three independent researchers (AM, JM, and AP) selected studies for inclusion with discrepancies resolved by discussion. The research was carried out with the use of the PRISMA flow diagram (Figure 1). The titles and abstracts were scanned, and potentially relevant studies were reviewed in full. We included eligible studies examining plasma or serum BDNF or IGF-1 levels in patients pre- and post-antidepressant treatment. Furthermore, each included study should make descriptions of the antidepressant use and time points in the therapy. Currently, there is no evidence that changes in the levels of neurotropic markers are associated with the clinical condition of a depressed patient. Therefore, the review includes only those studies in which the changes in the level of BDNF and IGF were determined—with the first measurement before treatment and the second after treatment. Clinical response was assessed using the Hamilton Depression Rating Scale (HAMD) and Montgomery–Asberg Depression Rating Scale (MADRAS) clinical scales, with clinical improvement was defined as a minimum of 50% symptom reduction post-treatment [51]. The selection criteria for publications to be included in the review were as follows: clinical diagnosis of MDD (without comorbidities); studies in which the BDNF and/or IGF-1 factors were assessed after the inclusion of antidepressants; assessment of markers during treatment with any of the following drugs: sertraline, paroxetine, fluoxetine, escitalopram, mirtazapine, vortioxetine, duloxetine, venlafaxine, agomelatine, milnacipran, duloxetine, tranylcypromine, amitriptyline, clomipramine, doksepine and moclobemid; at least two measurements of the marker during the study (at baseline and at the end of the study); and a subject age range of 18–65 years. Abstracts, case studies, family-based designs, population-based studies on healthy subjects, reviews and duplicate cohorts were excluded.

## 3. Results

### 3.1. BDNF

It is unclear whether low BDNF levels in patients with depression are primary or secondary. A possible explanation of reduced BDNF in depressed patients might reflect a genetic predisposition. Another hypothesis would be that stress-induced BDNF reductions might cause neuronal damage, which would in turn lead to acquired biological vulnerability. Further studies will be necessary in order to determine the precise mechanism underlying the relationship between reduced BDNF levels and the etiology of major depression [17,21]. Early studies conducted on rats assessing antidepressant responses showed that conventional antidepressant drugs, as well as electroconvulsive therapy (ECT), enhanced expression of BDNF and TrkB mRNA in the hippocampus and cortical regions in a timeframe similar to the onset of the antidepressant-like response [52,53]. Table 1 summarizes the research results on BDNF levels discussed in the present review (Table 1).

In our review, most of the data concerned the effects of SSRIs and SNRIs on neurotrophins due to the small number of studies conducted on other groups of antidepressants. Out of the few studies conducted on other classes of antidepressants, we also included those on MAOIs, TCAs and one study on ketamine.

Polyakova et al. meta-analyzed 21 (*n* = 735) studies concerning pre- and post-treatment serum and plasma BDNF levels according to treatment response in MDD patients [36]. Four studies included only the responders group, and six studies included both the responders and the non-responders groups. Six studies reported only the remitters group, one study included the remitters and non-responders groups and one study reported pooled data. The duration of the studies varied between 2 and 8 weeks. The meta-analysis divided patients into three subgroups depending on the response to antidepressants: remitters, responders or non-responders. The meta-analysis indicated that BDNF levels increased upon treatment in remitters (d = 0.85, 95% CI 0.39–1.29, *p* = 0.003, 7 effect sizes, *n* = 116) and responders (d = 1.33, 95% CI 0.69–1.97, *p* = 5.1 × 10^5^, 11 effect sizes, *n* = 252), whereas in the non-responders they remained stable (d = 0.15, 95% CI 0.33–0.63, *p* = 5.1 × 10^5^, 7 effect sizes, *n* = 118). The remitters and responders showed significantly higher serum BDNF levels than non-responders (*p* = 0.036 and *p* = 0.012, respectively). Studies concerning plasma BDNF changes were described as limited with no significant differences between subgroups. Overall, the meta-analysis reported increased BDNF levels for both serum (ES = 0.77, *p* = 1.5 × 10^6^) and plasma (ES = 0.30, *p* = 0.08) [36].

Arumugan et al. conducted meta-analysis concerning a total of 154 patients with MDD across six randomized control trials [38]. The duration of the included studies varied between 5 and 12 weeks. Different classes of antidepressants in different doses were used in the included studies. Four out of six articles divided patients in subgroups of responders and non-responders based on HAMD, including one that additionally was based on the BDNF level. The remaining two articles did not divide the group based on the response, but they evaluated the change in BDNF levels as well as HDRS scores. Three studies suggested a correlation between antidepressant treatment and BDNF concentration where the same was found to be increased. One study reported that different antidepressant drugs have variable effects on serum BDNF levels. Matrisciano et al. reported that different antidepressant drugs have variable effects on serum BDNF levels [55]. The study by Başterzi et al. did not reveal any significant [56] change, whereas a similar study by Hellweg et al. indicated a decline of BDNF by 12% in paroxetine-treated patients [57]. The study concluded that changes in BDNF levels do not occur uniformly for all the antidepressants [38].

Zhou et al. examined 20 studies to assess the effect of antidepressant drugs on the BDNF levels before and after treatment [37]. In total, 17 of the included studies investigated BDNF levels in serum and 5 investigated levels in plasma. A significant effect of antidepressant treatment on BDNF levels (SMD = 0.62, 95% CI = 0.31–0.94, Z = 3.92, *p* < 0.0001), as well as depressive symptoms amelioration with a significant decreased HDRS score (SMD = 2.78, 95% CI = 2.31–3.26, Z = 11.57, *p* < 0.00001) were observed with the use of random-effects model. The analysis indicated a significantly higher level of BDNF post-treatment in serum (SMD = 0.46, 95% CI = 0.20–0.72, Z = 3.43, *p* = 0.0006), which was not observed in plasma. Taking into account the length of the study, a statistically significant antidepressant effect was observed after 8 weeks of treatment (SMD = 0.71, 95% CI = 0.37–1.05, Z = 4.14, *p* < 0.0001). The study concluded that while both SSRIs and SNRIs could increase the BDNF levels after a period of antidepressant medication treatment, sertraline was superior to other three drugs (venlafaxine, paroxetine or escitalopram) in the early increase of BDNF concentrations with SMD 0.53 (95% CI = 0.13–0.93; *p* = 0.009) [38].

A randomized controlled trial by Brunoni et al. included patients with moderate depressive episode severity (defined as a Hamilton Depression Rating Score, 17 items (HDRS) ≥ 17) [20]. In the study treatment with sertraline (39 and 38 participants in the real and placebo arm, respectively, F1,153 = 0.78, *p* = 0.36), the drug did not change BDNF plasma levels over time according to clinical improvement (evaluated with the use of MADRS). The baseline BDNF levels were not associated with depression improvement [19]. Similarly, Chiou et al. did not observe a correlation between the serum BDNF levels and HDRS score in 41 drug-naïve first-episode MDD patients before and after 4 weeks of antidepressant treatment [39]. The antidepressants involved escitalopram (dose range: 10–20 mg/d), fluoxetine (dose range: 20–40 mg/d), mirtazapine (dose range: 30–60 mg/d), paroxetine (dose range: 20–40 mg/d) or venlafaxine (dose range: 75–225 mg/d). Response to treatment was defined as at least a 50% improvement in HDRS scores. The healthy controls had significantly higher serum BDNF concentration than patients with depression (F = 5.859, *p* = 0.017). Interestingly, the trend towards lower BDNF levels was observed in depressive women (F =3.334, *p* = 0.071), but not in depressive men (F = 2.896, *p* = 0.100). Post-treatment BDNF levels were not significantly elevated among 41 follow-up patients (10.7 ± 6.9 ng/mL vs. 12.9 ± 11.9 ng/mL; *p* = 0.126) and in the 28 responsive patients (12.0 ± 7.0 ng/mL vs. 15.3 ± 13.3 ng/mL; *p* = 0.113) [39].

Category B clinical trials, according to the classification by Siwek et al., were included in our review due to the small number of studies in category A [58]. In a study by Gupta et al., patients were assessed with HAMD and those with a score of ≥25 were treated with fluoxetine or agomelatine [40]. The assessment of BDNF levels took place before and after 12 weeks of treatment. In the fluoxetine group responders, HAMD and BDNF levels pre-treatment (30.83 ± 2.60 ng/mL and 2.54 ± 0.37 ng/mL respectively) significantly changed at 12 week (13.67 ± 1.79 ng/mL and 3.07 ± 0.33 ng/mL respectively). In the agomelatine group responders, the HAMD score and BDNF level at the start of treatment (31.1 ± 1.88 ng/mL and 2.44 ± 0.38 ng/mL, respectively) significantly changed (13.67 ± 2.22 ng/mL and 2.87 ± 0.44 ng/mL, respectively) post-treatment. Non-responders in both groups did not show any change in pre- and post-treatment BDNF levels. Troyan and Levada found that pre-treatment levels of serum BDNF (727.6 ± 87.9 pg/mL) were significantly lower in MDD patients in comparison to healthy controls (853.0 ± 93.9 pg/mL) (*p* < 0.0001) [41]. Vortixetine treatment significantly increased BDNF levels post-treatment; moreover, they were prominently higher than in healthy controls. (F = 9.36, *p* = 0.003). The psychopathological state of the patients was assessed with the use of MADRS and Clinical Global Impression (CGI). Vortixetine treatment improved all psychopathological and neuropsychological parameters and functioning [41].

The only study concerning intravenous infusions of ketamine was conducted by Zheng et al. [43]. The plasma BDNF levels were measured at baseline and after 13 and 26 days. Remission and response were defined as less than 10 points and a reduction of 50% or more in MADRS scores, respectively. Responders/remitters (11.0 ± 6.2/10.1 ± 5.8 ng/mL) had higher baseline BDNF levels than non-responders/non-remitters (8.0 ± 5.5/9.2 ± 6.4 ng/mL). A significant improvement in MADRS scores and pBDNF concentrations was found after completing six ketamine infusions compared to baseline (all *p* < 0.05). Baseline pBDNF concentrations were correlated with MADRS scores at 13 d (t = −2.011, *p* = 0.047) or 26 d (t = −2.398, *p* = 0.019) in depressed patients (all *p* < 0.05) [43].

### 3.2. IGF-1

Four studies of IGF-1 met the search criteria and were included in the present review. In a study conducted in a group of patients carrying the clinical diagnosis of MDD (*n* = 41) and a control group (*n* = 32), vortioxetine was given for 8 weeks [45]. Serum IGF-1 levels were determined at baseline (T0) and after 8 weeks of treatment (T1). The results showed that the baseline IGF-1 levels were significantly higher in MDD patients compared to the control group (*p* < 0.0001), while IGF-1 levels in the subsequent measurement (T1) in the MDD group were significantly lower than before the treatment (*p* < 0.0001) and not significantly different from HC (F = 1.86, *p* = 0.18). A positive correlation was found between IGF-1 levels and the diagnosis of MDD (r = 0.50, *p* < 0.01), the number (r = 0.43, *p* < 0.01) and duration of depressive episodes (r= 0.37, *p* < 0.01) and the severity of symptoms on the MADRS (r = 0.46, *p* < 0.01), and a negative correlation was demonstrated between IGF-1 and the results of cognitive function tests [41]. Similar results were reported by Levada et al. (2020). The vortioxetine treatment assessed in their study (*n* = 48) markedly decreased IGF-1 levels, which correlated with an improvement in clinical symptoms and cognitive function (*p* < 0.0001) [45]. Results of the research concerning IFG-1 levels is presented in Table 2.

In the study by Kopczak et al. IGF-1 levels were assessed at baseline and after 6 weeks of antidepressant treatment. The status of remission was defined by Hamilton depression rating scale (HAM-D) with a 21-item score <10. MMD patients had significantly higher IGF-1 levels at admission (*p* = 3.29 × 10^4^) and in week 6 (*p* = 0.002) compared to controls. In addition, baseline IGF-1 levels were significantly higher in non-remitters (*p* = 0.046) and there was a trend for higher IGF-I levels in week 6 (*p* = 0.11) compared to remitters. The study concluded that elevated IGF-1 levels were significantly associated with depression and impaired treatment response [46].

It has been observed that patients with MDD treated pharmacologically had significantly lower IGF-1 levels in comparison with the untreated group (B = −1.71 95% CI = −2.32 to −1.09, *p* < 0.001) [44]. Levels of IGF-1 showed significantly lower values in patients treated with fluoxetine (*n* = 52), citalopram (*n* = 96), paroxetine (*n* = 195), sertraline (*n* = 40), fluvoxamine (*n* = 38) and venlafaxine (*n* = 95) compared to the group not treated with antidepressants. No statistically significant correlation was observed for clomipramine (*n* = 26), amitriptyline (*n* = 21) and mirtazapine (*n* = 33). Patients in remission had higher IGF-1 levels compared to the healthy controls; however, this difference was not statistically significant (Cohen’s d = 0.08, *p* = 0.06) [44].

Described studies show that the authors noted a decrease in IGF-1 after the use of antidepressants in patients with clinical response to treatment. A decrease in IGF-1 levels has been observed with drugs such as vortioxetine, fluoxetine, paroxetine, citalopram, sertraline, fluvoxamine and venlafaxine. Results were inconclusive with amitriptyline.

Few studies attempting to determine the role of IGF-1 in the diagnosis and treatment of depression have been published. The role of IGF-1 in predicting the response to antidepressant drugs warrants further investigation. Data on the effects of antidepressant drugs on IGF-1 are very limited. Two studies evaluating the effect of vortioxetine and three studies on the use of other antidepressants have been included in the present review. These studies demonstrated a decrease in IGF-1 levels following treatment with vortioxetine [41,45]. Unfortunately, studies investigating the effect of other drugs on IGF-1 are lacking or have been conducted in an insufficient number of patients. A positive correlation has been demonstrated between IGF-1 levels and the diagnosis of MDD [41,45]. It should be, however, remembered that the studies were conducted on small groups of patients. Further human studies are necessary to determine IGF-1 usefulness in measuring response to antidepressants, as most of the data in medical databases concerns animal research. Summary of the results obtained in individual studies, including those included in meta-analysis, is presented in Table 3.

In the case of SSRI, four different medications (sertraline, escitalopram, paroxetine and fluoxetine) were used, eight studies reported increase of BDNF [21,40,54,58,59,60,61,62], one was ambiguous [55] and three did not report an increase of BDNF [19,39,56]. For SNRI, three drugs (vortioxetine, venlafaxine and duloxetine) were used, six reported increase of BDNF [41,42,54,59,63,65], one was ambiguous [64] and two did not report an increase (both of them used venlafaxine) [39,55]. All the results were measured at different time points. All studies on IGF-1 reported decrease of the marker post-treatment, regardless of the treatment used [41,44,45,46].

## 4. Discussion

BDNF is a growth factor synthesized in the cell bodies of neurons [67]. It affects neuronal maturation, formation of synapses and synaptic plasticity [68] BDNF is also associated with the development of psychiatric disorders. According to the neurotrophic hypothesis of depression, the deficiency of BDNF and other growth factors may contribute to the atrophy of certain limbic structures, including the hippocampus and prefrontal cortex, observed in patients with depression, and antidepressant drugs act by increasing the levels of BDNF [16,18,19,21]. IGF-1 has potent neurotrophic, neurogenic and neuroprotective effects. The activity of IGF-1 may be modulated by the immune system, whose confirmed role in the pathogenesis of depression is widely reported in scientific publications. These relationships are being investigated in further studies, which suggest that abnormal IGF-1 activity may be associated with the development of mood disorders [30].

Research shows that antidepressant therapy based on the regulation of monoamine neurotransmitters has some limitations [47,69,70,71]. No widely accepted biomarkers are available to assist diagnostics or treatment choice for individual patients. The timely selection of the optimal treatment for patients with depression is critical to improving remission rates [70]. Antidepressants that affect neural stem cells (NSCs) include selective norepinephrine reuptake inhibitors, monoamine oxidase inhibitors and serotonin reuptake inhibitors [72,73]. Studies confirming their involvement in neurogenesis showed an increase in the level of NSC proliferation after long-term use of fluoxetine, tranylcypromine and reboxetine [14]. During treatment, a 20%–40% increase in the number of new neurons was observed. The underestimated anti-inflammatory and antioxidant effect may be one of the potential mechanisms of action of the above-mentioned drugs [14].

The aim of our review was to update the knowledge on how antidepressants affect the level of neurotrophic markers (BDNF and IGF-1) and to assess whether the conclusions drawn in previous studies can be repeated after taking into account the studies included in the review. The review included four control trials [19,40,43,45], three meta-analyses [36,37,38] and two longitudinal studies [39,42] regarding BDNF levels (a total of 4643 people) and four clinical trials [41,44,45,46] on IGF-1 levels (3082 people) in plasma and/or serum before and after antidepressant treatment. In our review, we found that most of the studies conducted on serum BDNF [36,37,38,39,40,41] indicated that antidepressant treatment could increase BDNF concentration in remitters and responders. However, variety of drugs were used, including SSRI, SNRI and others, and different times of response were observed. Category A and B studies by Siwek et al. [54] concerning plasma BDNF [19,42] levels indicated that antidepressant treatment did not affect plasma BDNF levels. Treating treatment-resistant depression is an increasing problem; hence, this review includes the C category study of plasma BDNF levels before and after ketamine infusions [43]. Interestingly, the BDNF levels increased after an average time of 4 h after a low-dose infusion of ketamine, completely different from the evidence with other antidepressants, which require a more prolonged time [43]. However, there is not enough research and results of studies in different categories by Siwek et al. [54] and the available ones are inconsistent; therefore, it is difficult to draw conclusions. In the case of IGF-1, all reviewed studies reported its reduction as a result of antidepressant therapy [41,44,45,46]. However, the available data are limited (three studies on serum IGF-1 [41,45,46] and one on plasma IGF-1 [44]) and apply only to selected drugs; therefore, the association of IGF-1 changes with treatment response outcomes remains unclear.

Currently, there is still an insufficient number of human studies assessing the effect of antidepressants on the neurotrophic factors such as BDNF and IGF-1. Further research should focus on homogeneity of the research group and the determination of the optimal time for measuring BDNF and IGF-1 levels in plasma and serum. It is also necessary to continue research on how different drugs affect neurotrophins. Available data seem to be inconsistent in this regard.

## 5. Conclusions

Our review was aimed at updating the knowledge on the effect of antidepressant treatment on the levels of neurotrophic markers (BDNF and IGF-1) and to assess whether the conclusions drawn in previous meta-analyses can be repeated after considering the studies included in our review.

The use of the vast majority of antidepressive drugs in people with MDD affects the levels of neurotrophic factors. Lower baseline BDNF levels observed in patients possibly confirm an impairment of the stress-adaptation system and neuroplasticity in depression. In most cases, MDD patients experience a reduction in BDNF and increase in IFG-1 levels compared to healthy controls prior to treatment. Our review confirmed the conclusions of previous studies regarding the influence of drugs on the change in serum BDNF levels. The effect varied depending on the type of antidepressant drug used and the time of measurement. For plasma, studies showed no effect; however, there were too few studies to draw conclusions. Various types of antidepressants have a different effect on the BDNF levels; however, almost all of them increase serum BDNF. The possibility of using BDNF as a marker of antidepressant response and a new factor of targeted drug therapy is worth considering, but further research is needed. A review of 12 eligible studies found that BDNF levels were elevated after short-term (less than 12 weeks) use of any given antidepressant. The results are much better documented for BDNF in serum than in plasma, which may have good implications for further clinical trials. For IGF-1, only four studies met the inclusion criteria of this review. In all of them, a decrease in IGF-1 was observed during the use of antidepressants, but the data are insufficient to conclude that IGF-1 may be a predictor of response to treatment in depression. Further in-depth research on both factors seems to have very promising applications in understanding and treating depression.

## Figures and Tables

**Table 1:** Characteristic of the BDNF studies included in the present review.

Study	Year of Study	Grading System by Siwek et al. [54]	Type of Study	Patient Group (*n*)	Duration of Study (Weeks)	Markers	Medication	Influence of Drugs on Markers	Results
Polyakova et al. [36]	2014	A	Meta-Analysis	*n* = 2298	2–8	serum BDNF	Paroxetine, Escitalopram, Citalopram, Sertraline, Fluoxetine, Venlafaxine, Duloxetine Mirtazapine, Tranylcypromine, Amitriptyline, Clomipramine	YES	Serum BDNF was significantly decreased in patients in a depressive state compared with controls. The BDNF levels increased upon treatment in remitters and responders, whereas in the non-responders they remained stable. Serum BDNF changes in remitters and responders were significantly larger than in the non-responders.
Zhou et al. [37]	2017	A	Meta-Analysis	*n* = 633		serum and plasma BDNF	venlafaxine, paroxetine, sertraline, esciltalopram	+/−	Significant decreased HDRS score. Sertraline—statistically significant effect on the serum BDNF levels pre- and post-antidepressant treatment. Plasma BDNF—no effect.
Arumugam et al. [38]	2017	A	Meta-Analysis	*n* = 154	5–12	serum BDNF	Venlafaxine, Fluoxetine, Paroxetine, Citalopram, Sertraline, Milnacipran	+/−	Antidepressant therapy is associated with a change in BDNF level, but did not produce any significant impact on it. HDRS score signified a positive antidepressant treatment outcome.
Chiou et al. [39]	2017	A	Longitudinal study	*n* = 142 (drug-naïve first-episode MDD = 71 healthy controls = (71)	4	serum BDNF	escitalopram fluoxetine mirtazapine paroxetine venlafaxine	NO	The serum BDNF levels post-treatment were not significantly elevated.
Brunoni et al. [19]	2014	A	Randomized Controlled Trial	*n* = 103 (73 had their baseline and endpoint BDNF plasma levels analyzed)	6	plasma BDNF	sertraline,	NO	Sertraline did not change BDNF plasma levels over time and according to clinical improvement. There were no significant correlations between MADRS or HDRS scores changes with BDNF plasma changes.
Gupta et al. [40]	2017	B	Clinical Trial	*n* = 60 (agomelatine = 30 fluoxetine = 30)	12	serum BDNF	Fluoxetine, Agomelatine	YES	Agomelatine responders—serum BDNF level significantly increased at 12 weeks after treatment. Fluoxetine responders group—serum BDNF level significantly increased after 12 weeks of treatment.
Troyan & Levada. [45]	2020	B	Clinical Trial	*n* = 73 (treatment group = 30 healthy controls HC = 32 MDD = 41)	8	serum BDNF (sBDNF)	Vortioxetin	YES	BDNF levels were significantly higher post-treatment; moreover, they were prominently higher than in healthy controls (HC). Prominent inverse relationships was estabilished between BDNF concentrations and MDD status.
Sagud et al. [42]	2016	B	Longitudinal study	*n* = 88 (healthy controls = 44, MDD = 44)	4	plasma BDNF	Vortioxetine	YES	Vortioxetine treatment significantly increased plasma BDNF concentration in depressed patients compared to their baseline values.
Zheng et al. [43]	2021	C	Clinical Trial	*n* = 94	26 days	plasma BDNF	Ketamine	YES	Correlation and regression analyses showed significant associations between pBDNF concentrations at baseline and MADRS scores at 13 d and 26 d in depressed patients.

**Table 2:** Characteristic of the IGF-1 studies included in the present review.

Study	Year of Study	Grading System by Siwek et al. [54]	Type of Study	Patient Group (*n*)	Duration of Study (Weeks)	Markers	Medication	Influence of Drugs on Markers	Results
Troyan & Levada. [42]	2020	B	Clinical Trial	*n* = 73 (treatment group = 30 healthy controls HC = 32 MDD = 41)	8	serum IGF-1	Vortioxetin	YES	IGF-1 concentrations in MDD group post-treatment were significantly lower than pre-treatment and not significantly different from HC. Positive correlation was found between IGF-1 level and MDD status.
Bot et al. [45]	2016	B	Clinical Trial	*n* = 2714 (HC = 602)	2 years	Serum IGF-1	Fluoxetine Citalopram, Paroxetine, Sertraline Fluvoxamine, Venlafaxine, Clomipramine, Amitriptyline, Mirtazapine	YES	IGF-1 concentrations in MDD group post-treatment were significantly lower than pre-treatment and not significantly different from HC. Positive correlation was found between IGF-1 level and MDD status.
Levada et al. [46]	2020	B	Clinical Trial	completed = 48 (MDD = 78 HC = 47)	8	serum IGF-1	Vortioxetine	YES	Vortioxetine treatment significantly attenuated IGF-1 levels and improved all psychopathological and neuropsychological parameters. MDD patients had significantly higher serum IGF-1 levels than controls. IGF-1 had a good diagnostic value for predicting MDD in the whole sample with AUC. IGF-1 level has decreased after vortixetine treatment.
Kopczak et al. [47]	2015	B	Clinical Trial	*n* = 170 (MDD = 78 HC = 92)	6	Serum IGF-1	SSRI (selective serotonin reuptake inhibitors) SNRI (serotonin–norepinephrine reuptake inhibitor) TCA (tricyclic antidepressants) Mirtazapine	YES	IGF-I levels were significantly higher in patients at admission and in week 6 compared to HC.

**Table 3:** The effects of antidepressant drugs on BDNF and IGF-1.

Group of Antideoressants	Medication	Study	Duration of the Study (Weeks)	Patient Group (*n*)	Effect *
		BDNF			
SSRI	Sertraline	Wolkowitz et al. [21]	8	*n* = 10	↑
		Brunoni et al. [19]	6	*n* = 18	↓
		Umene-Nakano et al. [58]	8	*n* = 59	↑ (responders only)
		Matrisciano et al. [54]	6 months	*n* = 7	↑
		Gonul et al. [59]	8	*n* = 8	↑
	Escitalopram	Wolkowitz et al. [21]	8	*n* = 15	↑
		Matrisciano et al. [54]	6 months	*n* = 7	↑
		Aydemir et al. [60]	6	*n* = 20	↑
		Chiou et al. [39]	4	*n* = 2	↓
	Paroxetine	Yoshimura et al. [61]	8	*n* = 21	↑
		Gonul et al. [59]	8	*n* = 3	↑
		Chiou et al. [39]	4	*n* = 7	↓
		Hellweg et al. [56]	36 days	*n* = 20	↓
	Fluoxetine	Başterzi et al. [55]	6	*n* = 22	↑/↓
		Gupta et al. [40]	12	*n* = 30	↑
		Gonul et al. [59]	8	*n* = 5	↑
		Ghosh et al. [62]	12	*n* = 30	↑
		Chiou et al. [39]	44	*n* = 21	↓
SNRI	Vortixetine	Troyan et al. [41]	8	*n* = 30	↑
		Sagud et al. [42]	4	*n* = 44	↑
	Venlafaxine	Başterzi et al. [55]	6	*n* = 21	↓
		Matrisciano et al. [54]	6 months	*n* = 7	↑ (after 6 months of treatment)
		Katsuki et al. [63]	4	*n* = 48	↑
		Gonul et al. [59]	8	*n* = 10	↑
		Chiou et al. [39]	4	*n* = 8	↓
		Deuschle et al. (2012) [64]	28 days	*n* = 27	↔
	Duloxetine	Mikoteit et al. [65]	6	*n* = 21	↑
Other	Mirtazapine	Fornaro et al. [66]	12	*n* = 30	↑ early non-responders, ↔ early responders
		Deuschle et al. (2012) [64]	28 days	*n* = 29	↑
		Chiou et al. [39]	4	*n* = 3	↓
	Ketamine	Zheng et al. [43]	26 days	*n* = 94	↑
	Milnacipran	Yoshimura et al. [61]	8	*n* = 21	↑
	Amitryptyline	Hellweg et al. [56]	36 days	*n* = 20	↑
		IGF-1			
SSRI	N/A	Kopczak et al. [46]	6	*n* = 13	↓
	Fluoxetine Citalopram Paroxetine Sertraline Fluvoxamine	Bot et al. [44]	N/A	*n* = 52 *n*= 96 *n* = 195 *n* = 40 *n* = 38	↓ ↓ ↓ ↓ ↓
SNRI	N/A	Kopczak et al. [46]	6	*n* = 12	↓
	Vortioxetine	Troyan et al. [41]	8	*n* = 41	↓
		Levada et al. [45]	8	*n* = 48	↓
	Venlafaxine	Bot et al. [44]	N/A	*n* = 95	↓
TCA	Clomipramine Amitriptyline	Bot et al. [44]	N/A	*n* = 26 *n* = 21	↓ ↓ (not statistically significant)
	N/A	Kopczak et al. [46]	6	*n* = 14	↓
Other	Mirtazapine	Kopczak et al. [46]	6	*n* = 8	↓
		Bot et al. [44]	N/A	*n* = 33	↓ (not statistically significant)

* ↑ increase, ↓ decrease, ↔ unchanged.

## Data Availability

Not applicable.
